# Poverty, social exclusion and dental caries of 12-year-old children: a cross-sectional study in Lima, Peru

**DOI:** 10.1186/1472-6831-9-16

**Published:** 2009-07-07

**Authors:** Elsa K Delgado-Angulo, Martin H Hobdell, Eduardo Bernabé

**Affiliations:** 1 Unidad de Investigación en Salud Pública Dental, Departamento Académico de Odontología Social, Universidad Peruana Cayetano Heredia, Lima, Perú; 2 Department of Epidemiology and Public Health, University College London, London, UK

## Abstract

**Background:**

Socioeconomic differences in oral health have been reported in many countries. Poverty and social exclusion are two commonly used indicators of socioeconomic position in Latin America. The aim of this study was to explore the associations of poverty and social exclusion with dental caries experience in 12-year-old children.

**Methods:**

Ninety families, with a child aged 12 years, were selected from 11 underserved communities in Lima (Peru), using a two-stage cluster sampling. Head of households were interviewed with regard to indicators of poverty and social exclusion and their children were clinically examined for dental caries. The associations of poverty and social exclusion with dental caries prevalence were tested in binary logistic regression models.

**Results:**

Among children in the sample, 84.5% lived in poor households and 30.0% in socially excluded families. Out of all the children, 83.3% had dental caries. Poverty and social exclusion were significantly associated with dental caries in the unadjusted models (p = 0.013 and 0.047 respectively). In the adjusted model, poverty remained significantly related to dental caries (p = 0.008), but the association between social exclusion and dental caries was no longer significant (p = 0.077). Children living in poor households were 2.25 times more likely to have dental caries (95% confidence interval: 1.24; 4.09), compared to those living in non-poor households.

**Conclusion:**

There was support for an association between poverty and dental caries, but not for an association between social exclusion and dental caries in these children. Some potential explanations for these findings are discussed.

## Background

Among rich and poor countries, those people who are worse off in socioeconomic terms have worse health outcomes and higher mortality rates than those who are better off. However, it is not only the case that the poorest in society have poor health, but a gradient of ill-health and mortality exists across all socioeconomic groups [1-4]. This socioeconomic gradient has been found consistently also in different oral health measures around the world [5-10].

Poverty and social exclusion are two commonly used indicators of socioeconomic position in Latin America [11-13]. Absolute poverty refers to the lack of resources for survival and personal development as well as the necessary tools for relieving this situation [14,15]. People are considered to be poor when they cannot satisfy their basic needs [16]. Poverty imposes constraints on the material conditions of everyday life, by limiting access to the fundamental building blocks of health such as adequate housing, good nutrition and the opportunity to maintain optimal personal hygiene [2,4].

On the other hand, social exclusion refers to the accumulation of disadvantages, which isolate people from integrated social and physical human development [17,18]. Social exclusion prevents people from participating in education or training and gaining access to services and citizenship activities [18,19]. Being excluded from the life of society and being treated as less than equal, leads to worse health and greater risks of premature death [16,19]. Therefore, social exclusion refers not only to economic hardship, but also incorporates the process of marginalisation, that is, how individuals come to be excluded and marginalised in society [3,15]. Hence, social exclusion adds psychosocial aspects to the debate on poverty and links social disadvantages with individual participation and the stability of society [3,16,19].

Poverty, the extent of relative deprivation and the processes of social exclusion in a society have a major impact on the health of populations [2,3,16], and this also applies to oral conditions [6,20]. Parental socioeconomic position greatly affects the risk of dental caries in young children in both developed and developing countries [5,7,9,10,21].

According to national figures in Peru, about 40% of the population live in poverty [13,22], however, there are no reports on the extent of social exclusion. On the other hand, Peruvian children aged 12 years have moderate levels of dental caries [9], with an average of 3 decayed, missing and filled teeth per child [23,24]. Although some previous studies have explored the association between poverty and oral health in Latin-American children [21,25,26], there are no reports regarding the relationship of social exclusion to child oral health. The purpose of this study was to fill this gap. The study aimed, therefore, to explore the relationship between poverty, social exclusion and dental caries levels in 12-year-old Peruvian children.

## Methods

### Study sample

Ninety families, with a child aged 12 years, were selected from the 11 underserved communities linked to the Health Centre in Zapallal Alto (Lima, Peru), using a two-stage cluster sampling. There were 156 street blocks in these communities. For the sample selection, blocks were considered as clusters and chosen with a probability proportional to their size (i.e., the number of households per block). This selection procedure guaranteed that each household had an equal probability of selection. In each selected block, all households were screened for eligibility and those with a child aged 12 years were invited to participate. The design effect, defined as the ratio between the variances of the caries prevalence for cluster and simple random sampling [27], was 1.10, according to the pilot study. Therefore, the number of families required to estimate a significant association between poverty and dental caries prevalence (odds ratio of 3.0) with a statistical power of 80% and a type I error of 5% was 81, but this was increased to 89 to take account of the design effect. In all ninety-one families, in 43 blocks, were invited to participate.

Ethical approval was obtained from the International Research Board at the Universidad Peruana Cayetano Heredia. Only those children who agreed to participate and whose parents signed a consent form were enrolled in the study.

### Data collection

Information on poverty and social exclusion was collected during interviews with the heads of households. The socioeconomic position of each household was estimated using the Unsatisfied Basic Needs method [11,13,22]. This method is based on 5 dimensions: housing quality, household crowding, access to sanitation, access to education among minors and dependency rate (Table 1). These 5 indicators were added to form an index ranging from 0 to 5, where 0, and 1 to 5 were interpreted as non-poor and poor households, respectively. On the other hand, social exclusion was assessed through 11 indicators grouped into 3 domains: 6 indicators related to distributional and material aspects of exclusion, 4 related to relational and participatory aspects of exclusion and 1 related to long-term perspectives (Table 1) [19]. Each domain was considered as affected if one or more of its indicators were affected. A family was considered as socially excluded if it had all the 3 domains affected. Questions were translated and cross-culturally adapted following general recommendations [28-30].

**Table 1:** Sample distribution by indicators of poverty and social exclusion

**Variable**	**Domain**	**Indicator**	**n**	**(%)**
Poverty	Housing quality	Inadequate housing (walls and ceiling not made of bricks and cement)	72	(80.0)
	Household crowding	Overcrowded household (more than 3 persons per room)	4	(4.4)
	Access to sanitation	No access to safe water supply and sewerage facilities	90	(100.0)
	Access to education	Low school enrolment (1 or more children aged 6–12 not attending school)	2	(2.2)
	Dependence rate	High dependency rate (household head without secondary education, more than 2 dependants)	31	(34.4)

Social Exclusion	Distributional and material	Labour market performance (more than 12 months of unemployment)	2	(2.2)
		Living standards (last decile of Proportional Deprivation Index)	0	(0.0)
		Income poverty (below 50% of the mean equivalent household income)	13	(14.4)
		Educational status (No vocational training)	80	(88.9)
		Housing conditions (less than 1 room per person or no bath/toilet)	62	(68.9)
		Residential area (feeling of insecurity and bad living conditions in neighbourhood)	32	(35.6)
	
	Relational and participatory	Social relationships (no close friends and limited chances to contact other people)	12	(13.3)
		Politics (pessimism concerning political influence and no interest in politics)	82	(91.1)
		Anomie (feeling lonely or that life is too complicated)	27	(30.0)
		Anxiety (depression and frightening thoughts)	37	(41.1)
	
	Long-term perspective	Development of living conditions	28	(31.1)

Thereafter, children were dentally examined by a trained examiner (EKD), following the WHO recommendations [31]. Dental caries was diagnosed visually at the caries into dentine threshold and recorded as the number of decayed (D), missing (M) and filled (F) teeth or DMFT index. Intra- and inter-examiner reliability values at the end of the calibration process were 0.93 and 0.85 respectively (Generalised Kappa, p < 0.001 in both cases). A second visit for testing reliability during the main study was not conducted due to logistic reasons.

### Statistical analysis

The DMFT scores were dichotomised because of their skewed distribution in the sample. Children were considered as caries-free if they had a DMFT score of 0 and as having dental caries if they had a DMFT score higher than 0. Following this dichotomisation of the DMFT scores the unadjusted and adjusted associations of poverty and social exclusion with dental caries were assessed using binary logistic regression models. Study design was taken into account during the statistical analysis. Odds ratios (OR) were used to assess the strength of associations.

## Results

Ninety 12-year-old children (42 boys and 48 girls) participated in this study. The response rate was 98.9%. Of these children, 84.5% were living in poor households and 30.0% in socially excluded families. The distribution of the sample, according to each of the poverty and social exclusion indicators is shown in Table 1. The mean DMFT score was 3.93 teeth (SD: 3.72), ranging from 0 to 21 and the prevalence of dental caries was 83.3%.

As shown in table 2, poverty and social exclusion were found to be significantly associated with dental caries prevalence in the unadjusted models (p = 0.013 and 0.047, respectively). Children living in poor households and those in socially excluded families were 2.36 times (95% confidence interval: 1.20; 4.65), and 1.88 times (95% CI: 1.01; 3.51) more likely to have dental caries than those in wealthier households. However, the association between social exclusion and dental caries was no longer significant in the adjusted model (p = 0.077). On the other hand, children living in poor households were 2.25 times more likely to have dental caries after controlling for social exclusion and sex (95% CI: 1.24; 4.09).

**Table 2:** Binary logistic regression models for the associations of poverty and social exclusion with dental caries prevalence in 12-year-old children (n = 90).

**Explanatory variables**	**Unadjusted associations**			**Adjusted associations**		
	
	**Odds ratio**	**(95% CI)**	**p value**	**Odds ratio**	**(95% CI)**	**p value**
*Sex*						
Girls	1.00			1.00		
Boys	1.01	(0.52; 1.91)	0.985	1.04	(0.55; 1.96)	0.909
*Poverty (1 or more unsatisfied basic needs)*						
Non-poor	1.00			1.00		
Poor	2.36	(1.20; 4.65)	0.013	2.25	(1.24; 4.09)	0.008
*Social exclusion (three domains affected)*						
Integrated	1.00			1.00		
Excluded	1.88	(1.01; 3.51)	0.047	1.79	(0.94; 3.43)	0.077

## Discussion

This study examined the associations between poverty, social exclusion and dental caries of 12-year-old Peruvian children. The findings suggest that children living in poor households had more chance of having dental caries, even when account is taken of their social exclusion. However, the findings did not support an association between social exclusion and dental caries, as this association was completely attenuated when taking poverty into account. There are three potential explanations for these findings:

The first, possible explanation is that material deprivation really does have a greater impact on oral health among these children, compared to that of social exclusion. Previous research has shown the detrimental effects of being excluded from society on health. This evidence comes mainly from developed countries [4,16]. Wilkinson has suggested that up to a certain point per capita Gross Nation Income (GNI) does matter, but that beyond a certain level of per capita GNI, material living standards are no longer the main determinant of individuals' health [32,33], but rather income disparity between families. Although Peru has achieved sustainable economic development during the last decade, poverty levels remain as the main cause of morbidity and mortality in all age groups [13]. Similar findings have been reported in Brazil, where areas with the lowest levels of caries experience were concentrated in regions with better profile of social conditions [21,25]. Indeed, the mean number of persons per room in a household was the factor most strongly associated with dental caries levels [25]. So in the case of Peru it may still be that per capita GNI may still be the main factor in the levels of 12-year-old dental caries.

A second explanation relates to the potential mediating role of social exclusion in the relationship between poverty and dental caries. Material living conditions, where children live, may affect their participation in roles, relationships, functions, rights and responsibilities implied by membership of society, which in turn might affect their oral health. This explanation is supported by the significant unadjusted associations of poverty and social exclusion with dental caries and the 8%-decrease in the odds ratio for the association between poverty and dental caries after controlling for social exclusion. However, poverty and social exclusion were not significantly related in this sample (data not shown, p = 0.376), which fails to fulfil all the four conditions required to support a mediated pathway (i.e., the explanatory variable should be significantly related to the potential mediator and the outcome, the potential mediator should be related to the outcome and the relationship between the explanatory variable and the outcome should be attenuated when controlling for the potential mediator) [34,35]. Undoubtedly, further longitudinal studies are required to disentangle the roles of poverty and social exclusion in oral health.

The last but not least explanation relates to a certain degree of overlap between the constructs of poverty and social exclusion, as they were operationalised in this study. The assessment of the distributional and material aspects of social exclusion included indicators of long-term unemployment, income poverty, education, housing quality and neighbourhood conditions, which are strongly correlated to material deprivation, and therefore, tend to cluster around poor households [19]. This is supported by the fact that social exclusion remained significantly associated with dental caries, while accounting for poverty, when the material domain was left out in defining social exclusion (data not shown, p = 0.040). However, material indicators are of great value for gaining insights into the correspondence between insufficient living conditions in objective terms and their subjective evaluation, and also, in the context and conditions, under which such an evaluation takes place [19].

From explanations to limitations – as in any study there were limitations to this study, which need to be discussed. First, the data were cross-sectional. Causality cannot be determined using a cross-sectional design. Second, although this study used a random sample with an excellent participation rate, families were not representative of the entire Peruvian child population. Thus, the present findings are not generalisable beyond the study population. Third, the sample only included families living in underserved communities, and thereby, it did not capture the entire spectrum of socioeconomic conditions in Peru. Using a sampling frame of underserved communities may have restricted the variability in the sample and the statistical power to detect associations. However, sample size was based on conventional calculations and as such adequate for the aim of this study. Therefore, further studies, using a sampling frame that includes a more varied group of households, are needed to validate these findings. Fourth, socioeconomic position was indicated by poverty and social exclusion and not by the conventional measures of education, income or occupation [14,36]. However, the latter indicators are not reliable measures of poverty in low-income countries as they exclude other elements of deprivation [37,38]. Fifth, ORs were used for estimation in order to keep analyses straightforward and comprehensible. Recently, there has been much interest in estimating prevalence ratios (PRs) instead of ORs, especially in studies involving common outcomes [39,40]. However, there is still a debate about which measure to use [41] and what is the best approach to estimate PRs since results can be quite different depending on the method used [39,40,42]. Sixth, no attempt to control for oral health-related behaviours was carried out. As the aim was to assess the overall impact of socioeconomic position on dental caries levels of 12-year-old children, it was considered inappropriate to adjust for behaviours. Indeed, oral health-related behaviours are considered as merely intermediates of the relationship between socioeconomic indicators and oral health [20,43,44].

## Conclusion

Children living in poor households were about twice as likely to have dental caries after accounting for social exclusion than children living in wealthier households. On the other hand, social exclusion was not related to dental caries in children when account was taken of poverty. Some explanations for these findings are: first, the impact of material deprivation on dental caries in these children, compared to that of social exclusion is real; second, the potential mediating role of social exclusion in the relationship between poverty and dental caries; and third, the overlap between some poverty and social exclusion indicators masks the contribution made by either poverty or social exclusion separately. More studies using broader sampling frames are required to confirm the present findings.

## Competing interests

The authors declare that they have no competing interests.

## Authors' contributions

EKDA conceived of the study, collected data and drafted the first version of the manuscript. MH critically revised the manuscript. EB performed statistical analysis and also critically revised the manuscript. All the authors read and approved the final version of the manuscript.

## Pre-publication history

The pre-publication history for this paper can be accessed here:


